# Probabilistic cost-effectiveness analysis of controlled ovarian stimulation with recombinant FSH plus recombinant LH vs. human menopausal gonadotropin for women undergoing IVF

**DOI:** 10.1186/s12958-018-0386-2

**Published:** 2018-07-18

**Authors:** F. S. Mennini, A. Marcellusi, R. Viti, C. Bini, A. Carosso, A. Revelli, C. Benedetto

**Affiliations:** 1 Economic Evaluation and HTA (CEIS- EEHTA) - Faculty of Economics, University of Rome “Tor Vergata”, Via Columbia, 2, 00133 Rome, Italy; 2 Institute for Leadership and Management in Health - Kingston Hill Campus, Kingston Hill, Kingston upon Thames, KT2 7LB UK; 3 Gynecology and Obstetrics I, Physiopathology of Reproduction and IVF Unit, Department of Surgical Sciences, University of Torino, S. Anna Hospital, Via Ventimiglia 3, 10126 Torino, Italy; 4 LIVET Infertility and IVF Clinic, Via Tiziano Vecellio, 3, 10126 Torino, Italy

**Keywords:** Real world data, Retrieved oocytes, Cost-utility, QALYs

## Abstract

**Background:**

The association of recombinant FSH plus recombinant LH in 2:1 ratio may be used not only to induce ovulation in anovulatory women with hypogonadotropic hypogonadism but also to achieve multiple follicular developments in human IVF. The aim of this analysis was to estimate the cost-effectiveness of Controlled Ovarian Stimulation (COS) with recombinant FSH (rFSH) plus recombinant LH (rLH) in comparison with highly purified human menopausal gonadotropin (HP-hMG) in the woman undergoing in vitro fertilization (IVF) in Italy.

**Methods:**

A probabilistic decision tree was developed to simulate patients undergoing IVF, either using r-FSH + r-LH or HP-hMG to obtain COS. The model considers the National Health System (NHS) perspective and a time horizon equal to two years. Simulations were reported considering the number of retrieved oocytes (5–9, 10–15 and > 15) and transition probabilities were estimated through specific analyses carried out on the population of 848 women enrolled in the real-life.

**Results:**

The model estimated that patients undertaking therapeutic protocol with r-FSH + r-LH increase the general success rate (+ 6.6% for pregnancy). The incremental cost-effectiveness ratio (ICER) per quality-adjusted life year (QALY) of r-FSH + r-LH was below the willingness to pay set at €20,000 for all the considered scenarios.

**Conclusions:**

The cost-utility analysis demonstrated that the r-FSH + r-LH is a cost-effective option for the Italian National Health System (NHS).

## Background

The twentieth century witnessed the discovery of pituitary gonadotropins follicle-stimulating hormone (FSH) and luteinising hormone (LH) that were made available as medication after the extraction and purification from the urine of menopausal women [1]. The combination of urinary FSH (u-FSH) and human chorionic gonadotropin (u-hCG), a placental hormone displaying LH activity, has been available for the last forty years under the name of “human Menopausal Gonadotropin” (hMG) About twenty-five years ago, genetic engineering developed recombinant FSH (r-FSH) and recombinant LH (r-LH) by inserting the corresponding human DNA into Chinese hamster cells and then extracting and purifying the final molecules from their supernatant [2, 3]. Nowadays a highly purified hMG (HP-hMG, Meropur, Ferring Germany) has been introduced.

Urinary-derived and recombinant gonadotropins have been widely used to treat women with infertility due to chronic anovulation [4], or to provide a therapeutic stimulation to spermatogenesis [5]. In most cases, however, these are administered to obtain the so-called Controlled Ovarian Stimulation (COS), that is the multiple follicular developments aimed at getting the number of oocytes needed to perform in vitro fertilization (IVF). Several studies, as well as systematic reviews, have compared the effectiveness of urinary gonadotropins (u-FSH or HP-hMG) vs. r-FSH for COS, showing the superiority of r-FSH over u-FSH [4, 6, 7] and a substantial equivalence of r-FSH and HP-hMG [8–13]. Also, only in few countries the combination of r-FSH + r-LH in a 2:1 ratio (Pergoveris, Merck, Germany) for the treatment of patients with hypogonadotropic hypogonadism [14], is also licensed for COS. Comparative studies vs. hMG are rare and substantial do information are still missing [15, 16]. To date, the largest study comparing r-FSH + r-LH vs. HP-hMG in human IVF was conducted retrospectively on real-life data from clinical practice that were obtained in the IVF Unit of a University Hospital and in a private IVF Clinic [17]. In this context the r-FSH + r-LH association was demonstrated: (1) as effective as HP-hMG when the retrieved oocytes were less than 4, slightly (but not significantly); (2) superior when the retrieved oocytes were 5–8 and(3) significantly more effective in terms of pregnancy rate per embryo transfer (PR/ET) when they were 9 or more [17]. Moreover, in the same study, the advantage of using the r-FSH + r-LH therapy was even more pronounced when only mature oocytes were considered [17]. In support of the mentioned results, a multivariate logistic regression model confirmed that both the use of recombinant gonadotropins and the number of retrieved oocytes were increasing significantly the probability of a pregnancy, with an odds ratio (OR) of 1.628 and 1.083, respectively [17].

In another paper, a comparative analysis of legal restrictions on access to IVF was conducted in 13 EU countries. This study demonstrated as countries with the most generous public financing scheme tended to restrict access to IVF to a greater degree. Contrarily, no link was established between IVF utilization and the manner in which coverage was regulated or the level of public financing was set [18]. As a result of that, regulations seem generally more restrictive compared to the eligibility criteria in order to limit, through the reduced size of the covered population the budgetary outlays [18].

Nowadays the cost of pharmacological therapies represent a major issue [19] at international level and it is often considered as important as the effectiveness. Based on these premises, in the present study we aimed at performing a pharmaco economic analysis to estimate the cost-effectiveness of COS with r-FSH + r-LH in comparison with HP-hMG, considering the effectiveness of r-FSH + r-LH or HP-hMG (number of pregnancy, Positive or negative hCG test, Clinical pregnancy, Miscarriage, Cycle with embryo freezing, and Dropout) in terms of the Quality Adjusted Life Years (QALYs).

## Methods

### Model

**Fig. 1:**
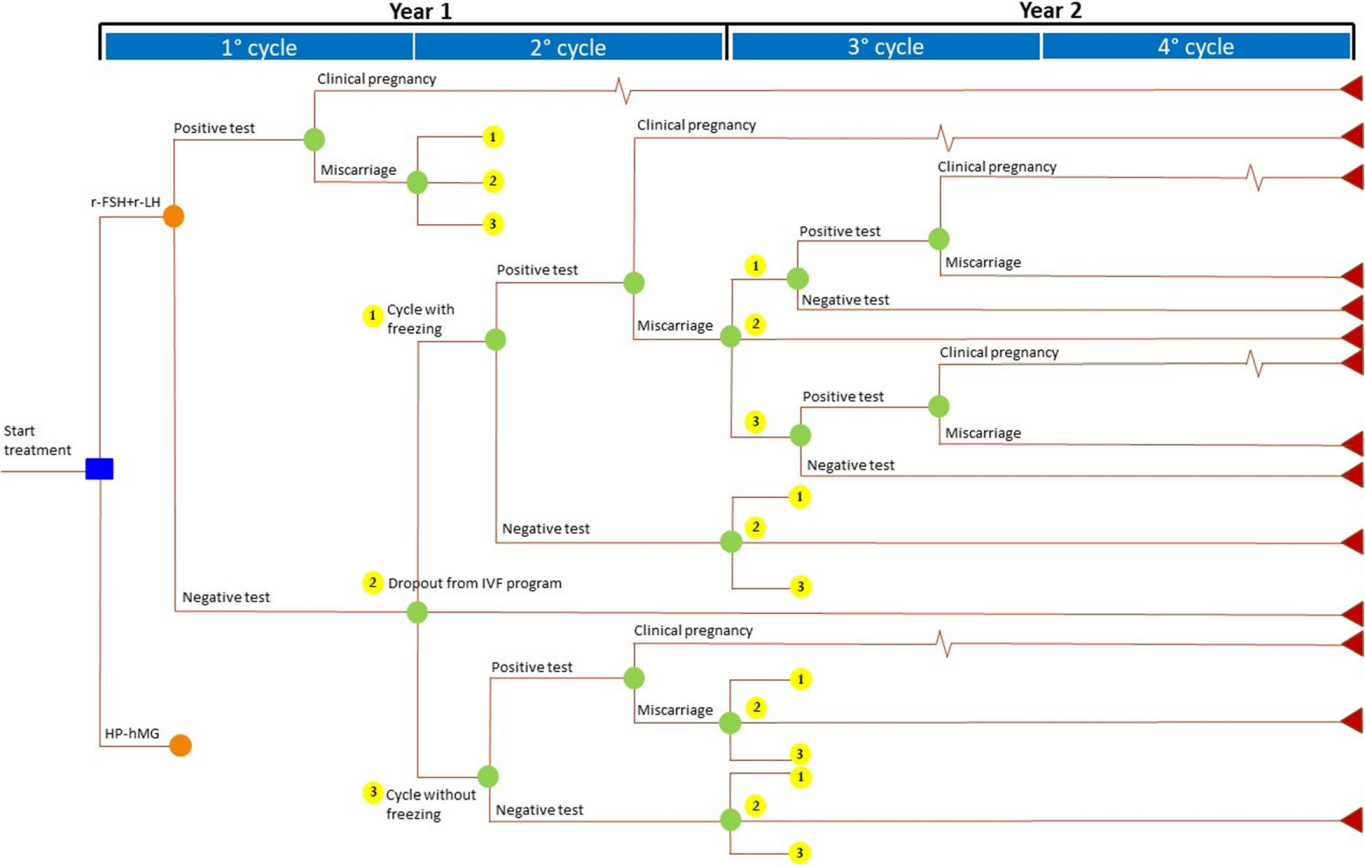
Cost Utility model structure – Decision tree

A probabilistic decision tree was developed to simulate the therapeutic path of two homogeneous cohorts of 1000 patients undergoing IVF, either using r-FSH + r-LH or HP-hMG and to obtain COS. Also, the pharmacological treatment was analyzed (Fig. 1). The study was performed in 2017.

The outcomes considered in the analysis were: (a) urinary hCG pregnancy test (performed 15 days after embryo transfer), (b) clinical pregnancy (foetal heartbeat at transvaginal ultrasound performed 2–4 weeks after an hCG+ test), (c) miscarriage (absence of foetal heartbeat at transvaginal ultrasound in a patient with hCG+), and (d) dropout from IVF program. The time horizon considered in the simulation was assumed to be equal to two years, with a simulation of one IVF cycle every 6 months for a maximum of 3 cycles. It was coherent with the literature [18] that underline how the most developed countries established public financing coverage limits for three or four cycles [18]. In Italy, most couples are submitted to a maximum of three IVF attempts during a two-year period, after which they can continue to undergo IVF, but without any reimbursement by the healthcare system.

In detail, once the COS with r-FSH + r-LH or HP-hMG had started, the patient was considered to have a different probability of incurring in a positive or negative hCG test according to the results previously published by Revelli et al. [17]. In case of a positive pregnancy test, the patient could have an US-detectable clinical pregnancy, in turn becoming an ongoing pregnancy or a miscarriage within the third month of pregnancy. In case of a negative test, the patient could undergo an additional IVF cycle using or not frozen embryos or decide to abandon the therapeutic program (dropout). The model assumes that patients without frozen embryos repeated the same COS used in the previous fresh cycle.

The population considered in the model was the one previously described by Revelli et al. [17], but we carried out sub-analyses grouping patients in a different way, and the following groups were created: 5–9 retrieved oocytes, 10–15 retrieved oocytes, and > 15 retrieved oocytes.

In order to estimate the QALYs of the two patient populations undertaking the therapeutic path with r-FSH + r-LH or HP-hMG, the utilities reported in Table 1 were considered. Due to the lack of precise information on the impact of various events on the quality of life of patients undergoing COS and IVF, the annual utility measures used in the model were obtained through the opinion of IVF experts.

**Table 1:** Utilities considered in the model

Therapeutic path	Utility	Months	Source
r-FSH + r-LH *(MIN - MAX)*	0.8 *(0.72–0.88)*	1	Expert opinion
HP-hMG *(MIN - MAX)*	0.8 *(0.72–0.88)*	1	Expert opinion
Positive hCGtest *(MIN - MAX)*	0.9 *(0.81–0.99)*	3	Expert opinion
Negative hCGtest *(MIN - MAX)*	0.7 *(0.63–0.77)*	5	Expert opinion
Clinical pregnancy *(MIN - MAX)*	1 *(0.90–1.00)*	2	Expert opinion
Miscarriage *(MIN - MAX)*	0.5 *(0.45–0.55)*	2	Expert opinion
Cycle with embryo freezing *(MIN - MAX)*	0.9 *(0.81–0.99)*	1	Expert opinion
Cycle without embryo freezing *(MIN - MAX)*	0.8 *(0.72–0.88)*	1	Expert opinion
Dropout *(MIN - MAX)*	0.4 *(0.36–0.44)*	6	Expert opinion

The utilities reported in Table 1 and obtained through the support of expert clinicians were transformed into monthly utilities to perform the analysis.

Each monthly utility was applied and weighed according to the reference period reported in Table 1. The two COS protocols were considered to have a priori identical impact on the quality of life.

### Transition probabilities

The probability of obtaining a positive hCG test and, later on, having or not a miscarriage was obtained through specific analyses carried out on the population of 848 women enrolled in the real-life, previously published in a clinical study [17]. Such probabilities were calculated stratifying the population of patients according to the type of COS therapy and to the number of retrieved oocytes (Table 2). The statistical significance was evaluated with a chi-square test and Fisher’s exact test when appropriate.

**Table 2:** Transition probabilities used in the model

	r-FSH + r-LH	HP-hMG	*p*-value for comparison	Source
≥ 5 Oocytes				
Positive hCG test *(MIN - MAX)*	0.34 *(0.31–0.37)*	0.23 *(0.21–0.25)*	0.007	Analyses from [17]
Negative hCG test *(MIN - MAX)*	0.66 *(0.60–0.73)*	0.77 *(0.69–0.84)*		
Clinical pregnancy *(MIN - MAX)*	0.84 *(0.76–0.92)*	0.82 *(0.74–0.90)*	0.790	
Miscarriage *(MIN - MAX)*	0.16 *(0.15–0.18)*	0,18 *(0.16–0.19)*		
5–9 oocytes				
Positive hCG test *(MIN - MAX)*	0.28 *(0.26–0.31)*	0.22 *(0.20–0.24)*	0.158	Analyses from [17]
Negative hCG test *(MIN - MAX)*	0.72 *(0.65–0.79)*	0.78 *(0.70–0.86)*		
Clinical pregnancy *(MIN - MAX)*	0.82 *(0.74–0.90)*	0.79 *(0.71–0.86)*	0.646	
Miscarriage *(MIN - MAX)*	0.18 *(0.16–0.19)*	0.21 *(0.19–0.24)*		
10–15 oocytes				
Positive hCG test *(MIN - MAX)*	0.43 *(0.39–0.48)*	0.24 *(0.22–0.27)*	0.025	Analyses from [17]
Negative hCG test *(MIN - MAX)*	0.57 *(0.51–0.62)*	0.76 *(0.68–0.83)*		
Clinical pregnancy *(MIN - MAX)*	0.88 *(0.80–0.97)*	0.87 *(0.78–0.95)*	1.000	
Miscarriage *(MIN - MAX)*	0.12 *(0.10–0.13)*	0.13 (*0.12–0.15)*		
> 15 oocytes				
Positive hCG test *(MIN - MAX)*	0.56 *(0.50–0.61)*	0.33 *(0.30–0.37)*	0.202	Analyses from [17]
Negative hCG test *(MIN - MAX)*	0.44 *(0.40–0.49)*	0.67 *(0.60–0.73)*		
Clinical pregnancy *(MIN - MAX)*	0.80 *(0.72–0.88)*	1.00 *(0.90–1.00)*	0.524	
Miscarriage *(MIN - MAX)*	0.20 *(0.18–0.22)*	0.00 *(0.00–0.00)*		
Transition probabilities valid for all the sample				
Cycle with embryo freezing *(MIN - MAX)*	0.30 *(0.27–0.33)*	0.30 *(0.27–0.33)*		Expert opinion
Cycle without embryo freezing *(MIN - MAX)*	0.40 *(0.36–0.44)*	0.40 *(0.36–0.44)*		Expert opinion
Dropout *(MIN - MAX)*	0.30 *(0.27–0.33)*	0.30 *(0.27–0.33)*		Expert opinion

Analysing the general characteristics of the patients, it was observed that by increasing the patients’ age, the number of retrieved oocytes would decrease. In particular, the average age of the patients (36.7 years for the whole sample) was 36.4 for women with less than 5 retrieved oocytes, 36.6 for women with 5–9 retrieved oocytes, 36 for women with 10–15 retrieved oocytes, and 35.6 for women with more than 15 retrieved oocytes. Therefore, the model assumed that the probability of obtaining a positive hCG pregnancy test was likely to decrease with age with an odds ratio of 0.901 (95% CI: 0.863–0.940) [17].

The transition probabilities of obtaining embryo freezing as well as the probability of dropping-out from the IVF program were assumed to be independent from the number of retrieved oocytes, independent from the medication used for COS, and were estimated through the expertise of clinical practice.

### Cost parameters

The costs considered in the model refer only to the direct costs covered by the Italian NHS for IVF treatment (Table 3). For the cost estimates related to pregnancy and miscarriage, we referred to the range of fees of hospital health care for acute patients [20]. Specifically, assuming that 60% of patients would have a vaginal delivery and the remaining 40% a caesarean section (expert opinion based on the general trend in Italy for IVF patients), such cost was obtained through a weighted average of the Italian diagnosis-related group (DRG) 371 (cesarean section without complications) and 373 (vaginal delivery without complications) rates. As far as miscarriage is concerned, the cost was obtained as a simple average between DRG 376 (spontaneous miscarriage without surgery) and 377 (spontaneous miscarriage with dilatation and curettage) rates.

**Table 3:** Input data to calculate costs

Medications	Dose (IU)	Formulation (IU)	Ex-factory price	Source
r-FSH + r-LH	2453.46	150	€ 72.55	[27]
HP-hMG	2801.49	75	€ 16.10	[27]
Pre-treatment tests	Frequency		Unit cost	Source
Hysterosalpingography	1		€ 116.10	[14], expert opinion
Transvaginal ultrasound	1		€ 45.90	[14], expert opinion
Gynaecological consultation	1		€ 21.30	[14], expert opinion
Serum oestradiol (E2)	1		€ 14.30	[14], expert opinion
Follicle-stimulating Hormone (FSH)	1		€ 11.90	[14], expert opinion
Fertility test of the seminal fluid	1		€ 7.90	[14], expert opinion
Luteinising Hormone (LH)	1		€ 12.90	[14], expert opinion
Prolactin (PLR)	1		€ 12.70	[14], expert opinion
Thyroid-stimulating Hormone (TSH)	1		€ 12.40	[14], expert opinion
Free thyroxine (FT4)	1		€ 12.60	[14], expert opinion
Free triiodothyronine (FT3)	1		€ 12.70	[14], expert opinion
Blood samples drawing	1		€ 2.70	[14], expert opinion
Tests during each IVF cycle	Frequency		Unit cost	Source
Transvaginal ultrasound	3		€ 45.90	[14], expert opinion
Gynaecological consultation	3		€ 21.30	[14], expert opinion
Serum oestradiol (E2)	3		€ 14.30	[14], expert opinion
Blood samples	3		€ 2.70	[14], expert opinion

*IU* International Unit

The COS therapy medication cost was estimated on the ex-factory price, that is the price set at the level of the manufacturer, net of the discounts provided by law, the formulation and the total dose observed in the reference study [17]. The cost of the monitoring before and during COS was derived from the work of Papaleo et al. [14] integrated by indications supplied by expert opinions (Table 3).

### Cost-effectiveness analysis

Cost-effectiveness analysis as applied to health economics provides an approach to medical decision making [21]. A cost-effectiveness analysis is a type of economic evaluation in which cost is expressed over some unit of benefit (life years gained, symptom free months, etc.) [21]. A cost-utility analysis is a type of cost-effectiveness analysis in which the benefit is expressed in utility [21]. The most commonly used measure of benefit in a cost-utility analysis is the QALY [21].

Quality-adjusted life years (QALYs) has become increasingly used as a healthcare outcome measure and as an integral part of cost-utility analysis [22, 23]. It combines length of life and quality of life into a single index number [24]. It is calculated as the area under the curve when measuring utility over time [22]. The utility can be thought of as the preference for a particular health state: the greater the preference, the greater the utility associated with it [22]. Health state utilities are used to quantify health-related quality of life and are ranked on a scale 0–1, with 0 being equivalent to death and 1 being a state of perfect health. Health state utilities measured over time can be used to generate QALYs by multiplying the duration in a particular health state by the utility associated with that state.

Most health conditions lie somewhere in between, although it is possible for the lower bound to have a negative value [25]. The effectiveness of r-FSH + r-LH vs HP-hMG was expressed has incremental QALY gained. Results were expressed as an incremental cost-effectiveness ratio (ICER). Mathematically, it can be described as ICER = (C1 − C2)/(E1 − E2), where C1 and E1 are the cost and effect in the intervention or treatment group and where C2 and E2 are the cost and effect in the control care group [21].

### Sensitivity analysis

In order to consider the variability of the results based on the model parameters, two sensitivity analyses were conducted.

The first one (*deterministic analysis*) used a one-way deterministic approach in which the model results were obtained changing one parameter of the model at a time, based on the variability found in the literature or assumed by the authors. In this specific case, the following scenarios were considered:

- probability to undergo an IVF cycle without embryo freezing (base case = 0.4): Min = 0, Max = 1;
- probability to dropout from the therapeutic program (base case = 0.3): Min = 0, Max = 1;
- change of transition probabilities based on the assumed variability of ±5% compared to the base case (Table 2);
- change of utilities associated with different health conditions of a given patient based on an assumed variability of ±5% compared to the base case (Table 1);
- change of pregnancy and miscarriage costs (pregnancy base case = € 1600.00, miscarriage base case = € 1525.50): Min pregnancy (DRG 373) = € 1272.00, Max pregnancy (DRG 371) = € 2092.00; Min miscarriage (DRG 376) = € 1264.00, Max miscarriage (DRG 377) = € 1787.00;
- probability to undergo an IVF cycle with embryo freezing (base case = 0.3): Min = 0, Max = 1.
- follow-up (base case = 2 years): 1, 2 and 3 IVF cycles.

The second analysis (*probabilistic analysis*) was conducted using a probabilistic sensitivity approach [Probabilistic Sensitivity Analysis (PSA)], modeling all the parameters through Montecarlo simulations, each of them according to a specific probabilistic distribution. The probabilistic distribution was chosen applying what is generally reported for the development of the probabilistic models in the economic evaluations, distinguishing between costs (gamma distribution) and epidemiological parameters (beta distribution) [19].

The results of the deterministic analysis were presented through a tornado chart, while the results of the probabilistic analysis were presented through the Cost-Effectiveness Acceptability Curve (CEAC).

## Results

### Epidemiological results

Based on the model simulations, the patients undergoing a therapeutic protocol with r-FSH + r-LH had a lower time to pregnancy (TTP) than the women receiving HP-hMG (7.2 vs. 7.5 months for positive hCG test and 13.2 vs. 13.5 months for clinical pregnancy, respectively) (Table 4). Furthermore, the general success rate over the time horizon established in the analysis (2 years) was higher for patients treated with r-FSH + r-LH, compared to HP-hMG, both in terms of positive hCG test (28.2% vs. 20.6%, respectively) and of clinical pregnancy (23.6% vs. 17.0%, respectively) (Table 4).

**Table 4:** Epidemiological parameters from 1000 patients’ simulation – patients having at least 5 retrieved oocytes

	HP-hMG	r-FSH + r-LH
Cycle 1 clinical pregnancies	191	284
Cycle 2 clinical pregnancies	97	128
Cycle 3 clinical pregnancies	51	60
Clinical pregnancies	339	473
*Average time at clinical pregnancy (months)*	*13.5*	*13.2*
*Clinical pregnancy rate*	*17.0%*	*23.6%*
	HP-hMG	r-FSH + r-LH
Cycle 1 positive hCGtest	232	339
Cycle 2 positive hCG test	118	153
Cycle 3 positive hCG test	62	72
Positive hCG tests	413	563
*Average time at positive hCG test (months)*	*7.5*	*7.2*
*Positive hCG test rate*	*20.6%*	*28.2%*
	HP-hMG	r-FSH + r-LH
Miscarriages	37	45

**Fig. 2:**
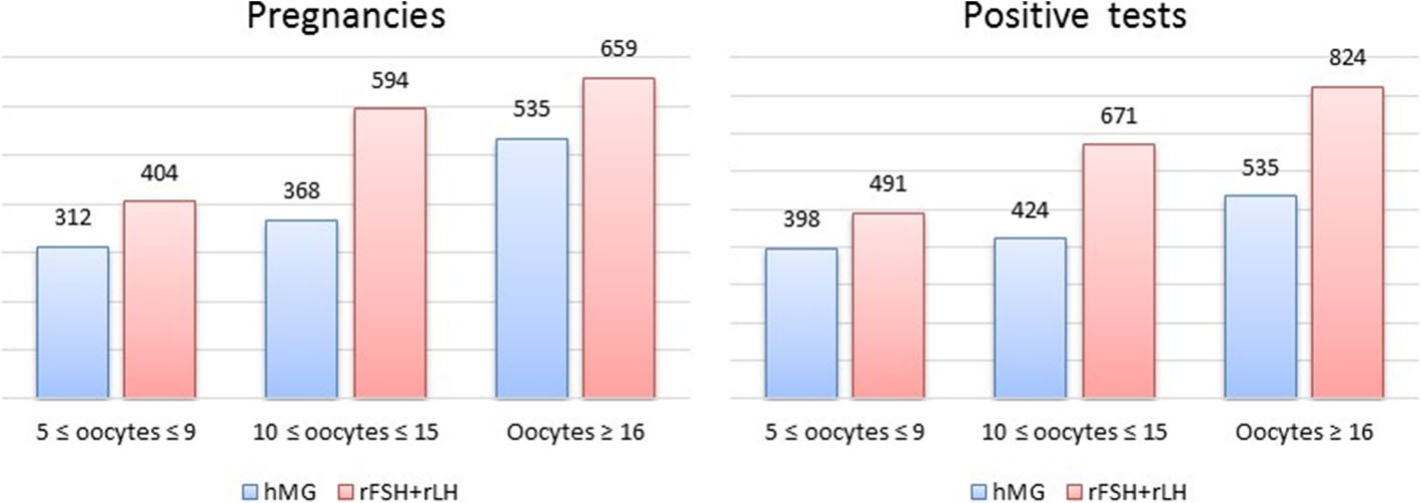
Epidemiological parameters from simulations on patients stratified according to the number of retrieved oocytes

These data were also confirmed after the stratification of the results according to the number of retrieved oocytes when patients with at least 5 oocytes were considered (Fig. 2).

### Cost-effectiveness results

Table 5 reports the results in terms of quality of life (QALYs) and costs for each ongoing pregnancy (clinical pregnancy without miscarriage) in the NHS perspective.

**Table 5:** Average cost and effectiveness results per 1000 patients – 2 years base case follow-up (patients with ≥5 retrieved oocytes)

	Medication cost	Monitoring cost	Miscarriage/pregnancy cost	Overall cost	Pregnancies	Cost per pregnancy
HP-hMG	€ 889,012	€ 967,508	€ 654,635	€ 2,511,155	339	€ 7400
r-FSH + r-LH	€ 1,690,582	€ 899,956	€ 894,236	€ 3,484,774	473	€ 7375
*Difference*	+€ 801,570	-€ 67,552	+€ 239,601	+€ 973,618	*+ 133*	-€ 25

In particular, the cost of the drug and miscarriage/pregnancy resulted to be higher in the r-FSH + r-LH scenario (+€ 801,570 and + € 239,601 respectively), whereas the monitoring cost was lower (−€ 67,552). This was due to two main factors: (a) the number of pregnancies using the r-FSH + r-LH approach was higher than with HP-hMG, with a higher absolute cost, but a lower cost per pregnancy (€ 7375 vs € 7400 respectively) and (b) the higher number of pregnancies and hCG positive tests involved a higher cost for the NHS with an improvement of the quality of life of over 4 QALYs gained for 100 women (Table 6).

**Table 6:** Cost-effectiveness table per number of retrieved oocytes (average results per treated patient)

	Cost	QALYs	Incremental Cost	Incremental QALYs	ICER per QALYs
≥ 5 oocytes					
HP-hMG	€ 1256	0.71			
r-FSH + r-LH	€ 1742	0.76	€ 487	0.04	€ 11,365
5 ≤ oocytes ≤ 9					
HP-hMG	€ 1254	0.70			
r-FSH + r-LH	€ 1726	0.73	€ 472	0.03	€ 16,309
10 ≤ oocytes ≤ 15					
HP-hMG	€ 1254	0.72			
r-FSH + r-LH	€ 1751	0.80	€ 497	0.08	€ 6569
≥ 16 oocytes					
HP-hMG	€ 1272	0.78			
r-FSH + r-LH	€ 1824	0.82	€ 551	0.04	€ 12,274

With reference to the cost-effectiveness, Table 6 reports the average cost-utility values per patient obtained for the whole time horizon considered in the analysis, stratified according to the number of retrieved oocytes. The results indicate that, at the end of the analyzed period, the ICER per QALY values were below a willingness to pay of € 20,000 – 40,000.

### Sensitivity analysis results

**Fig. 3:**
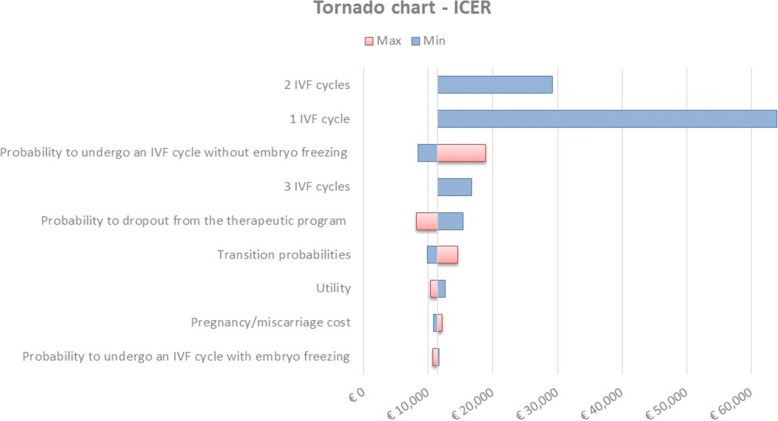
Deterministic sensitivity analysis – Tornado chart. *ICER* Incremental Cost-Effectiveness Ratio

Figure 3 reports the results of the one-way deterministic sensitivity analysis, for patients with ≥5 retrieved oocytes. The parameters mostly influencing results are represented by the variation of the transition probabilities concerning the possibility for the patient to undergo an IVF cycle without embryo freezing and to quit the therapeutic program. In all the considered scenarios, the ICER values never exceeded € 20,000 per QALY gained, showing a good robustness of the results.

**Fig. 4:**
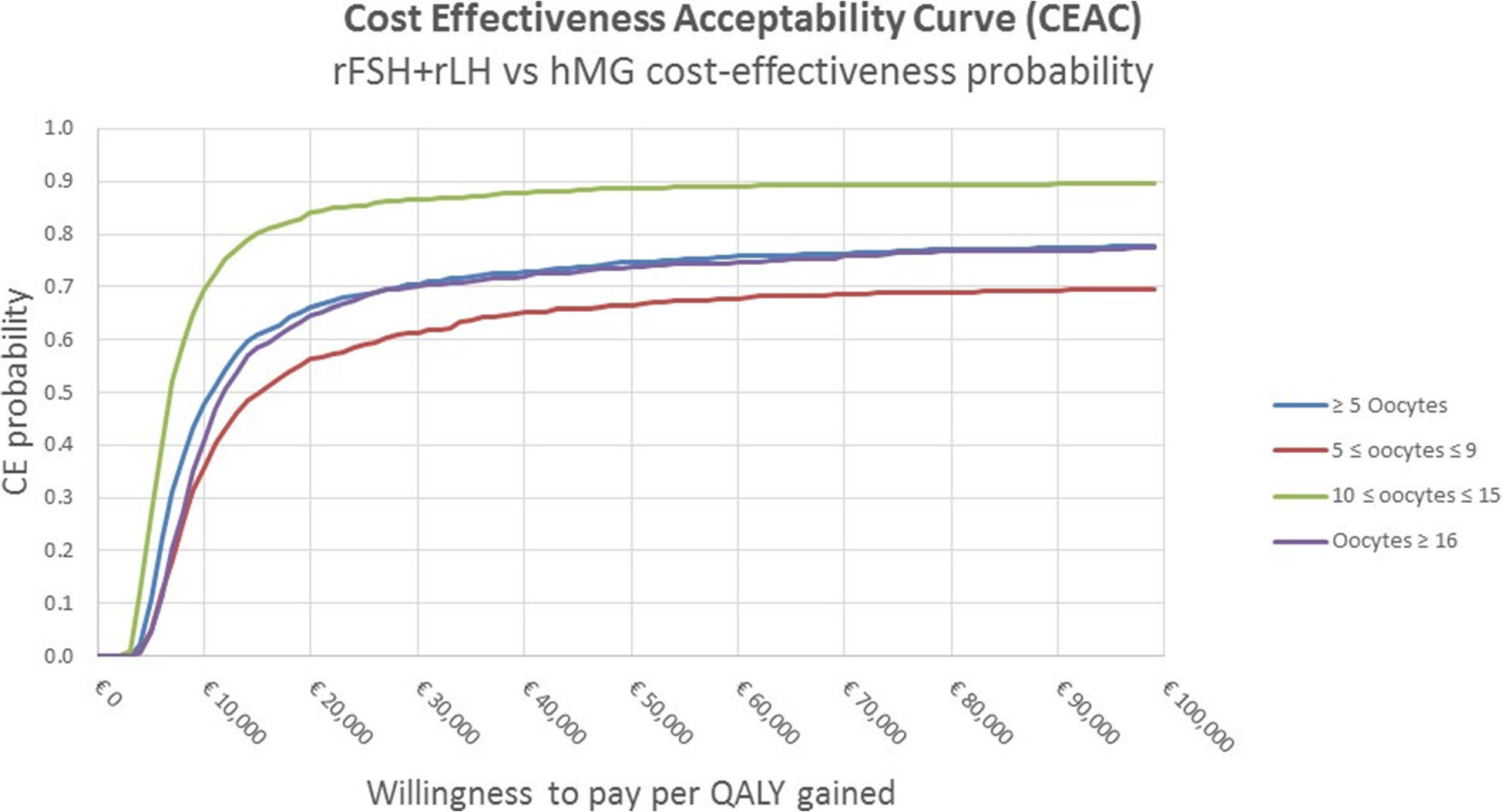
Cost-effectiveness Acceptability Curve per number of retrieved oocytes. *CE* Cost Effectiveness

The probabilistic sensitivity analysis conducted according to the number of retrieved oocytes, corresponding to the population of patients with 10–15 retrieved oocytes, confirmed that at the end of the observation period the r-FSH + r-LH therapeutic protocol was cost-effective compared with HP-hMG. Considering a willingness to pay of about € 15,000, the probability that the r-FSH + r-LH therapeutic protocol could result to be advantageous with respect to HP-hMG resulted to be higher than 80% (Fig. 4).

**Fig. 5:**
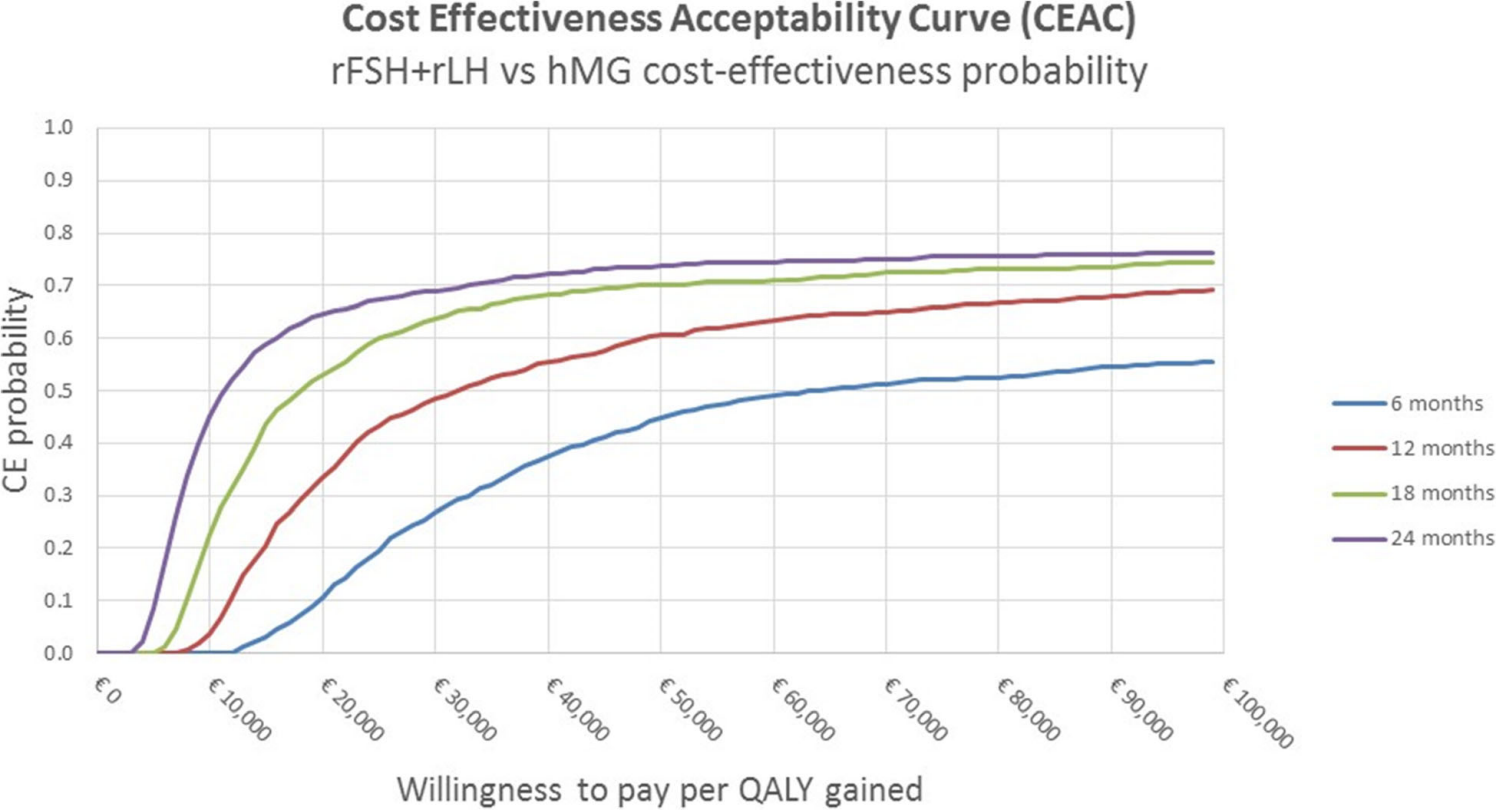
Cost-effectiveness Acceptability Curve – ≥ 5 retrieved oocytes. *CE* Cost effectiveness

The results of the probabilistic analysis reported in Fig. 5 confirm the above calculation, showing higher uncertainty in the short term (blue line). As the time horizon - and consequently the number of IVF cycles - extends, the cost-effectiveness probability increases. At the end of the observation period, with a willingness to pay of about € 35,000, the r-FSH + r-LH therapeutic protocol appeared to be the most cost-effective with a probability higher than 70%.

## Discussion

The association of recombinant FSH plus recombinant LH in 2:1 ratio (Pergoveris, Merck, Germany) may be used not only to induce ovulation in anovulatory women with hypogonadotropic hypogonadism [14], but also to achieve multiple follicular developments in human IVF. To date, there are still scarce or poorly informative data that compared the COS using r-FSH + r-LH with that obtained using other medications (e.g.HP-hMG) in the IVF setting. Indeed in several studies [8–13] the urinary-derived HP-hMG was compared to r-FSH alone, without taking into account that HP-hMG contains not only FSH, but also LH and hCG that act on the same receptor of LH and have a powerful LH activity.

A randomized controlled trial (RCT) comparing HP-hMG and r-FSH + r-LH in patients undergoing IVF included only 122 women and showed comparable outcomes in terms of pregnancy rate [16]. Unfortunately, this study was clearly underpowered as it was designed to assess the difference in oocyte rather than in the pregnancy rates. Another RCT study on 579 patients, included patients submitted to intrauterine insemination (IUI) rather than to IVF [15]. This showed comparable outcomes, but without enough observations to show a significant difference of effectiveness between medication. In fact, IUI allows to get a much lower pregnancy rate than the expected when using IVF in patients of the same age (17.3% in the cited study [15] vs. approximately 30–40%).

To the best of our knowledge, the wider study comparing HP-hMG and r-FSH + r-LH in IVF patients is a retrospective analysis of real-life data that included 848 women classified as expected “poor” or “normal” responders to gonadotropins [17]. Data were collected under real-life practice circumstances in IVF Unit in S. Anna Hospital (Torino, Italy), and the pregnancy rate with fresh embryo transfer was calculated by stratifying patients according to the number of retrieved oocytes, in order to exclude that a difference in the results could be due to oocyte availability [17]. The study showed an improvement in pregnancy rate according to the increasing number of retrieved oocytes. However, when comparing patients within the same oocyte, the two medications obtained comparable results when up to 4 oocytes, and a slight/ not significant advantage with 5–8 oocytes. However, the PR/ET became significantly higher when 9 or more oocytes were available [17]. The multivariable logistic regression analysis confirmed that both the use of r-FSH + r-LH and the total number of retrieved oocytes increased the probability of pregnancy, with an odds ratio (OR) of 1.628 and 1.083, respectively, showing that the medication used for COS was even more influent than the number of oocytes itself.

Despite the contribution yielded by the previous literature in the field, the cost of COS, is still a major issue in times of global economic restrictions. This is relevant both in health systems where the patients cover the costs with their own resources and in countries where the National Health Service (NHS) takes care, partially or completely, of the expenses. Due to the evidenced gap of knowledge and the relevance of the proposed subject, the present study represents the first attempt to evaluate the cost-effectiveness of COS when recombinant gonadotropins in 2:1 combination (Pergoveris) or HP-hMG are used in a large series of patients undergoing IVF. Precisely we aimed to measure, through a sophisticated economic analysis the overall cost-effectiveness of IVF cycles based on the previously published database [17].

As a result, the present analysis demonstrates that r-FSH + r-LH therapy showed higher cost-effectiveness than HP-hMG in the considered two-years observation periods with a slightly lower overall cost per pregnancy despite a higher cost per medication. The cost-effectiveness acceptability curve (CEAC) showed that the observed difference between the two medications was likely to further increase if the time horizon was prolonged and the number of IVF cycles rose. The advantage given by recombinant gonadotropins vs. HP-hMG was not linked to a higher number of retrieved oocytes because calculations were performed after patients’ stratification in subgroups and having the same number of available oocytes. In detail, for the population of patients obtaining 5 or more oocytes, the r-FSH + r-LH therapy resulted to be highly cost-effective compared with HP-hMG, with an ICER period equal to €11,365 per QALY. Interestingly the patients that obtained the highest advantage from being treated with r-FSH + r-LH instead of HP-hMG were those with 10–15 retrieved oocytes, who had an ICER of € 6569 per QALY. According to the medical literature, patients getting 10–15 retrieved oocytes are among those with the best prognosis after fresh embryo transfer [26] and may be considered “normal responders” to gonadotropin stimulation, representing approximately 40–50% of the overall population undergoing IVF.

Our analysis, showed two main limitations: (a) the lack of reports on the quality of life of patients undergoing IVF did not allow an exact quantification of the utility measures used in the model, some of which were obtained considering the opinion of IVF experts; (b) the database on which our analysis was based did not report about embryo freezing and dropouts from the program. Even in this case, their incidence was estimated according to the opinion of IVF experts. However, the one-way sensitivity analyses allow to take this uncertainty into account, and therefore these two limits should not have affected the validity of our results. A final limitation, c) regards the generalisability of our results on other countries. This study was specifically settled on the Italian general practice and the model was populated with Italian costs and tariffs different than what happens in other European countries. However, the regulation in Europe demonstrates a difference in term of access to IVF [18]. Contrarily, no link was established between IVF utilization and the manner in which coverage was regulated or the level of public financing was set [18]. As a result of that, we can assume that the general management of these patients could be homogeneous around Europe and the model structure represents a general approach for other countries. Further analysis, could consider the same model structure adapting the specific economic parameters and evaluate the cost-effectiveness results for each country perspective. The National Healthcare Assistance in Italy covers the complete cost for gonadotropin treatment in IVF, provided that the patient is younger than 45 and her basal FSH circulating level is below 30 U/l. Not all countries have such a system, but in general, most healthcare systems help patients to face a percentage (variable in different countries) of the economic cost of IVF medications and procedure. Although the results found herein are not perfectly applicable to other countries due to such differences in the healthcare assistance reimbursement, the relative proportion of the cost of ovarian stimulation with recombinant FSH and LH vs. hMG is rather constant everywhere, and therefore the general concepts expressed in this study may be of interest even outside Italy.

The strength point of this analysis is that is based on real-world data [17]; the transition probabilities used to perform the model have been obtained from an Italian study which collected data from clinical charts of IVF Unit in S. Anna Hospital (Torino, Italy).

## Conclusions

In conclusion, the present cost-utility analysis demonstrated that the r-FSH + r-LH combination, although more expensive than HP-hMG when medication costs are considered, may be effectively used to obtain COS in IVF patients without increasing the overall costs for the patients or the NHS. On the contrary, the r-FSH + r-LH association allows getting slightly reduced costs for pregnancy, improved cost-effectiveness and quality of life, especially when the so-called “normal-responders”, who represent the majority of IVF patients, are considered.
